# Hypoglycemic Effect of Resveratrol: A Systematic Review and Meta-Analysis

**DOI:** 10.3390/antiox10010069

**Published:** 2021-01-07

**Authors:** Beatriz Isabel García-Martínez, Mirna Ruiz-Ramos, José Pedraza-Chaverri, Edelmiro Santiago-Osorio, Víctor Manuel Mendoza-Núñez

**Affiliations:** 1 Research Unit on Gerontology, FES Zaragoza, National Autonomous University of Mexico, Mexico City 09230, Mexico; isabelgm06@gmail.com (B.I.G.-M.); mirna1411@yahoo.com.mx (M.R.-R.); 2 Department of Biology, Faculty of Chemistry, National Autonomous University of Mexico (UNAM), Mexico City 04510, Mexico; pedraza@unam.mx; 3 Hematopoiesis and Leukemia Laboratory, Research Unit on Cell Differentiation and Cancer, FES Zaragoza, National Autonomous University of Mexico, Mexico City 09230, Mexico; edelmiro@unam.mx

**Keywords:** glucose, insulin, glycated hemoglobin, glycemic control, insulin resistance, polyphenolic compounds

## Abstract

Resveratrol (RV) is a polyphenolic compound with antioxidant, anti-inflammatory, and hypoglycemic properties. Several in vitro and animal model studies have demonstrated the beneficial effects of RV; however, the results in humans are not conclusive. After a search of different databases, 32 studies were selected for this systematic review and 30 were included in the meta-analysis. Studies that evaluated the effect of RV on glucose, insulin, HbA1c, and insulin resistance (HOMA-IR) levels were included. A significant decrease of glucose (−5.24 mg/dL, *p* = 0.002) and insulin levels (−1.23 mIU/L, *p* = 0.0003) was observed. HbA1c and HOMA-IR did not show significant changes. Due to heterogeneity, sub-analyzes were performed. Sub-analysis by dose revealed that glucose levels improve significantly after the administration of 500–1000 mg/day of RV (−7.54 mg/dL, *p* = 0.002), while insulin improves with doses lower than 500 mg/day (−1.43 mIU/L, *p* = 0.01) and greater than 1000 mg/day (−2.12 mIU/L, *p* = 0.03). HbA1c and HOMA-IR remained unchanged after sub-analysis by dose. Our findings suggest that RV improves glucose and insulin levels in subjects with type 2 diabetes mellitus (T2DM) and aged 45–59 years, regardless of the duration of the intervention. HbA1c improves with interventions ≥3 months. HOMA-IR does not exhibit significant changes after RV administration.

## 1. Introduction

Resveratrol (RV) is a polyphenolic compound that includes two benzene rings connected through a methylene, and three hydroxyl groups in its structure. This structure allows the RV molecule to give up electrons to distinct free radicals (FR) and thereby attenuates the damage to biomolecules. Moreover, RV has anti-inflammatory properties due to its ability to block the activation and subsequent translocation of nuclear factor κB (NFκB), which is responsible for the synthesis of pro-inflammatory proteins, such as tumor necrosis factor α (TNFα), interleukin 1 (IL1), interleukin 6 (IL6), and prothrombotic molecules [1,2,3]. RV is found in grapes, peanuts, and blueberries, although the plant *Polygonum cuspidatum* (Mexican baboo, knotty herb from Japan) is the main natural source of this compound. In the last two decades, multiple investigations have been carried out on the therapeutic properties of RV, which are given by the participation of RV in the signaling pathways that modulate the processes of apoptosis, mitochondrial dysfunction, platelet aggregation, oxidative stress, and inflammation [4,5]. In this sense, RV is an attractive compound for the adjunctive treatment of chronic noncommunicable diseases (NCDs), such as diabetes, cardiovascular diseases, arthritis, neurodegenerative disorders, and even cancer [6,7].

Regarding the therapeutic effects of RV, these are strongly related to the activation of sirtuin 1 (SIRT1) and AMP-activated protein kinase (AMPK). Both proteins act as energy regulators due to their participation in metabolism and mitochondrial function, which makes them a suitable target for the treatment of metabolic diseases, such as type 2 diabetes mellitus (T2DM) [7,8].

Scientific evidence, obtained from in vitro studies and in animal models, suggests that RV has antioxidant, anti-inflammatory, and even anti-cancer properties; however, the results of clinical trials are not conclusive. In this context, some clinical trials suggest that RV exerts beneficial effects on metabolic diseases (obesity, metabolic syndrome, and diabetes), which has been evidenced by its ability to reduce the levels of lipids, glucose, and some adipokines. Furthermore, it has been observed that after RV administration, the antioxidant capacity increases and the concentrations of pro-inflammatory markers decrease [9,10,11].

Despite the above, in some investigations carried out in humans, no evidence of the therapeutic effects of RV has been found. Therefore, there is currently no consensus regarding the therapeutic benefits of RV and the dose at which they are presented, so research on this compound is still continuing [12,13]. Considering this, the aim of this systematic review and meta-analysis is to show and analyze the findings on the hypoglycemic effect of different doses of RV from clinical trials and quasi-experimental studies.

## 2. Materials and Methods

The study was carried out according to the guidelines for the presentation of systematic reviews and meta-analyzes (PRISMA 2009) [14].

### 2.1. Search Strategy

A literature search was carried out in the following databases: PubMed-Medline; Scopus; Cochrane library; Web of Science; Wiley online library; ScienceDirect; and Lilacs. The search was carried out among all articles published from January 1980 to 31 May 2020. The following search strategy was used: Resveratrol AND (glycemic control OR fasting glucose OR insulin resistance). A National Autonomous University of Mexico (UNAM) thesis search was also carried out to identify unpublished studies that could potentially be included in the review. Titles and abstracts identified through the search strategy were independently assessed by two reviewers (B.I.G.-M. and M.R.-R.), and discrepancies were resolved by a third reviewer (V.M.M.-N.). Once the titles and abstracts that met the selection criteria had been selected, the full texts of potentially relevant articles for the review were retrieved and an exhaustive review was carried out to select the definitive studies.

### 2.2. Inclusion Criteria

The inclusion criteria were as follows: (a) Blind or double-blind randomized clinical trials (RCTs); (b) the use of RV as a nutritional supplement; (c) placebo controlled; (d) published in the English language; (e) evaluation of at least one of the following biochemical markers: Serum glucose and insulin levels; HbA1c; and insulin resistance (HOMA-IR); (f) duration of at least 2 weeks; and (g) the participation of adults aged ≥20 years, without the distinction of sex, healthy, or with metabolic and/or inflammatory conditions, except cancer.

### 2.3. Exclusion Criteria

The exclusion criteria were as follows: (a) Studies that administered RV in combination with other compounds (e.g., quercetin); (b) studies that administered compounds derived from resveratrol, red wine, or a diet rich in polyphenols; (c) studies without a control group; (d) pilot studies; and (e) research only available in summary, to avoid risk of bias given a lack of information.

### 2.4. Outcomes

The primary outcomes included the following:

- Serum glucose and insulin levels;
- HbA1c percentage;
- HOMA-IR value.

### 2.5. Data Extraction

Once the studies were chosen, two reviewers (J.P.-C. and E.S.-O.) performed data extraction. Data extracted for the systematic review included the first author’s last name, year of publication, study design, dose of resveratrol used, duration of intervention, sample size, characteristics of participants (age, health status, and/or metabolic condition), parameters evaluated, and findings of each study. For the meta-analysis, the following data were extracted: The means (Ⴟ) and standard deviations (SD) of the pre- and post-treatment measurements corresponding to the levels of glucose, insulin, HbA1c, and HOMA-IR. The Ⴟ, SD, mean difference, and their respective SD were calculated in cases in which the studies did not report them; for this purpose, appropriate statistical methods were used [15,16,17]. The Ⴟ was calculated from the median, the minimum and maximum values, and the sample size (n), using the formula Ⴟ = (minimum value + 2n + maximum value)/4. The SD was calculated with the formula SD = √1/12[Ⴟ2 + (maximum value − minimum value)2]. When standard error (SE) was reported, the formula SD = SE × √n was applied to calculate SD. For calculation of the mean difference, the following formula was used: Difference in means = meanpost-treatment − meanpre-treatment. Furthermore, for the calculation of the corresponding SD, the following formula was used:

SDdifference = √[(SDpre-treatment2 + SDpost-treatment2) − (2 × R × SDpre-treatment × SDpost-treatment)], where R = 0.8.

Because the studies did not report the data in the same units, some conversions were necessary. To convert mmol/L to mg/dL of glucose, the value was multiplied by 18. The conversion of insulin units from pmol/L to mIU/L was performed by dividing pmol/L ÷ 6945. To convert the units of HbA1c, the following formula was used: %HbA1c = (mmol/mol/10,929) + 2.15. In some cases, the HOMA-IR index was also calculated, for which the following formula was used: HOMA-IR = [insulin (mIU/L) × glucose (mg/dL)]/405.

### 2.6. Assessment of Risk of Bias and Quality of Studies

After retrieving the full text of the selected studies, they were reviewed in detail to eliminate those that did not meet the inclusion criteria, in addition to assessing their methodological quality. For this purpose, the Cochrane collaboration risk of bias assessment tool was used. This tool considers seven items for evaluation, including the generation of the random sequence, allocation concealment, blinding of staff and participants, blinding of the analysis of results, incomplete results data, selective reports of results, and other sources of bias.

### 2.7. Statistical Analysis

To estimate the overall effect of RV supplementation on glucose, insulin, HbA1c levels, and HOMA-IR values, a random effects model was used. This model considers intra- and inter-study heterogeneity. Heterogeneity was assessed using the I2 test, considering the existence of significant heterogeneity if I2 > 50%. Subgroup analyzes were performed, separating by resveratrol dosage (<500 mg/day, 500–1000 mg/day, and >1000 mg/day), health status (with T2DM and without T2DM), duration of the intervention (<3 months and ≥3 months), and age (<45 years, 45–59 years, and >60 years). In addition, sensitivity analyzes were performed to assess the effect of each study on the overall effect. For this, all meta-analyzes were carried out, removing one study at a time. Funnel plots and Egger’s test were performed to assess publication bias. The value of *p* < 0.05 was considered as statistically significant. The statistical analyzes were performed with Review Manager version 5.3 software from the Cochrane collaboration.

## 3. Results

### 3.1. Literature Search

**Figure 1:**
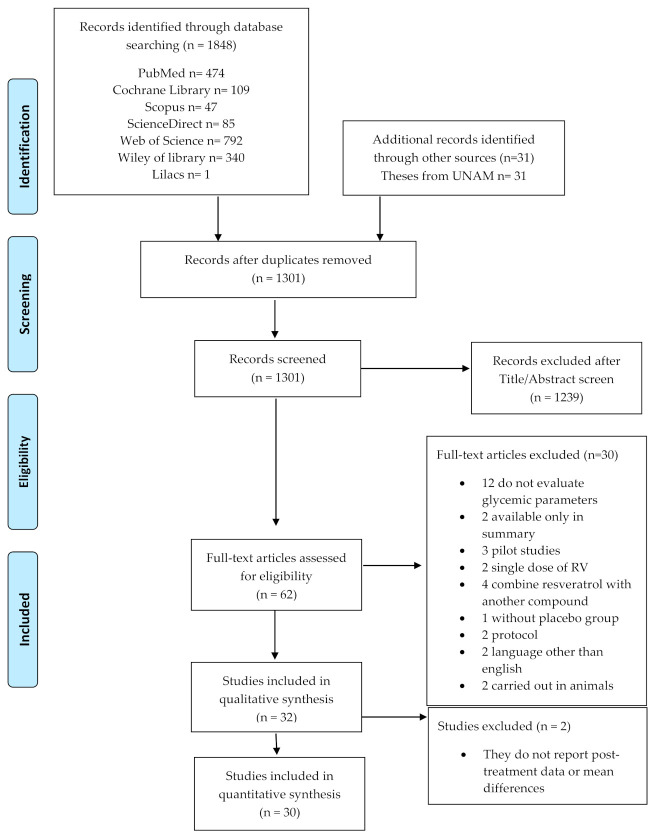
Flow diagram of study selection included in the systematic review and meta-analysis.

The total number of articles identified from the database search was 1848, plus 31 theses from UNAM. After reviewing the titles and abstracts, duplicate studies were eliminated, as well as those that did not meet the selection criteria, leaving a total of 62 eligible articles, of which the full text was recovered. After reviewing the full text of the 62 preselected studies, 30 of them were eliminated from the qualitative analysis for several reasons (see Appendix A), which are presented in Figure 1, and 32 studies were included in the systematic review. However, two studies (Bo et al. 2013 and Brasnyó et al. 2011) were discarded from the quantitative analysis because they did not present the necessary information (means before and after treatment or mean difference), so only 30 studies were included in the meta-analysis (Figure 1).

### 3.2. Study Characteristics

**Figure 2:**
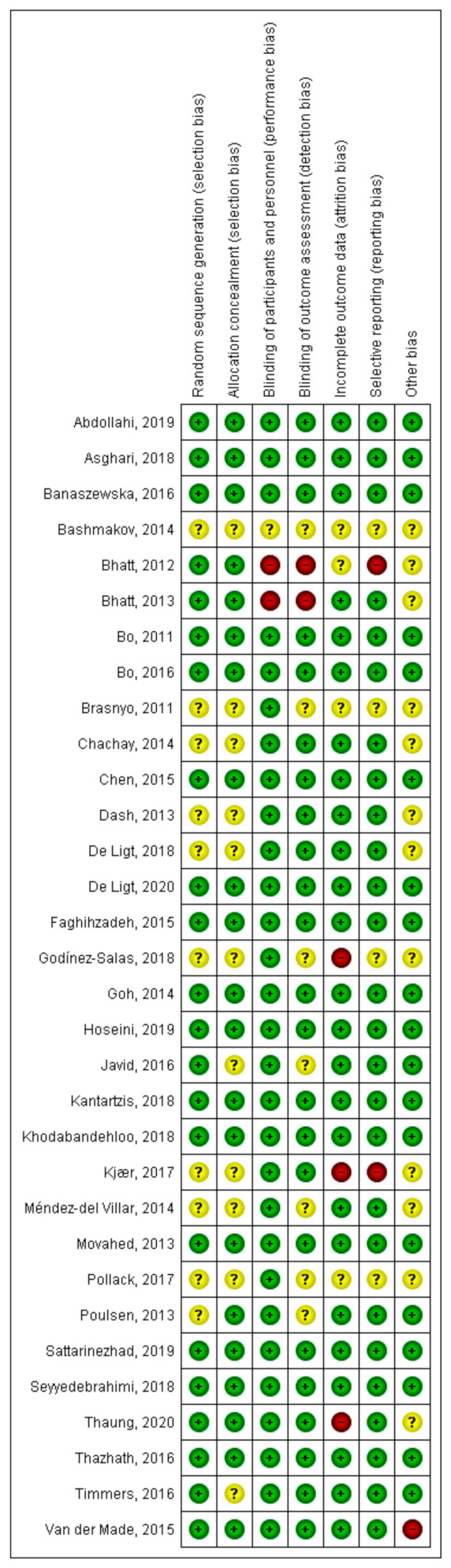
Assessment of the risk of bias and methodological quality of clinical trials included. Most clinical trials display a low risk of bias.

Of the included randomized clinical trials, 22 had a parallel double-blind design, 7 a double-blind crossover design, 1 was parallel-blind, and 2 were open-label. The total number of participants included in the meta-analysis was 1651 with different ages and health conditions. The subjects included in each study ranged from 8 to 129, the doses used ranged from 10 to 3000 mg/day, and the duration of the interventions was at least 4 weeks and a maximum of 12 months. The characteristics of the studies included in the present review are shown in Table 1. The results on the assessment of the methodological quality and risk of bias are presented in Figure 2.

### 3.3. Meta-Analysis

**Figure 3:**
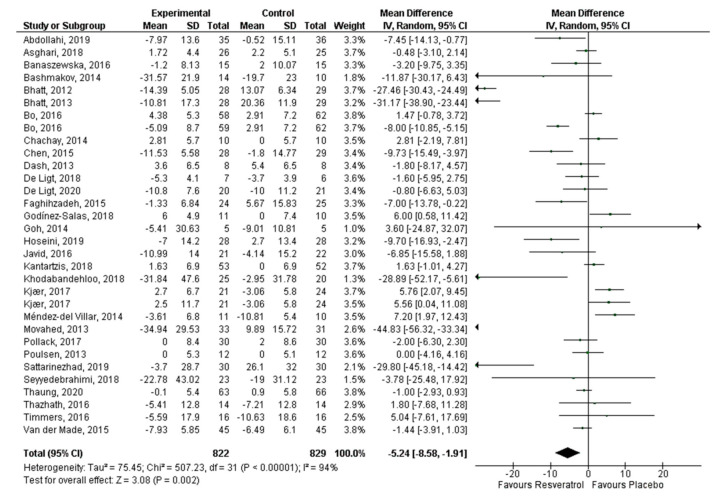
Effect of resveratrol on glucose levels.

**Figure 4:**
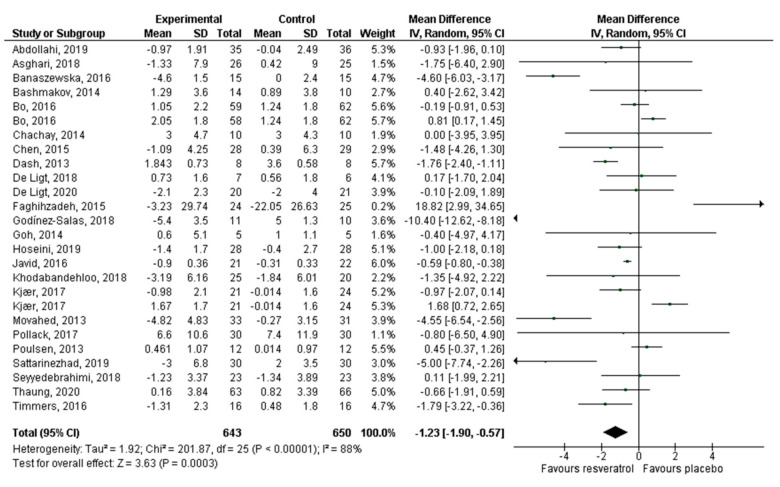
Effect of resveratrol on insulin levels.

**Figure 5:**
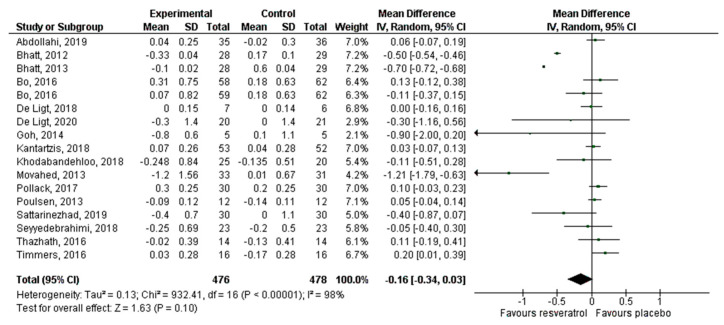
Effect of resveratrol on HbA1c.

**Figure 6:**
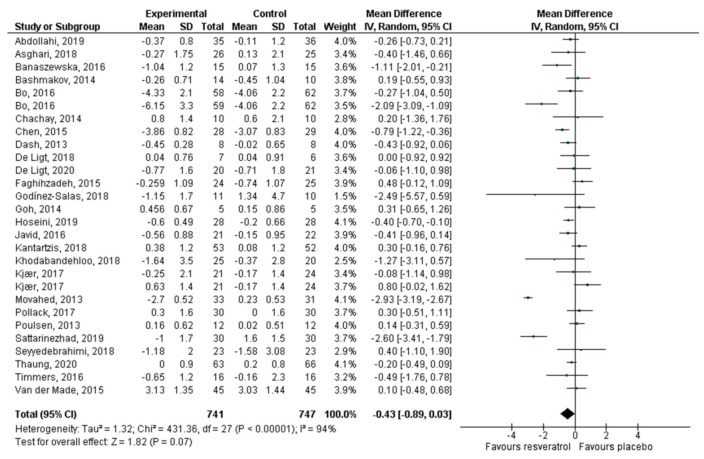
Effect of resveratrol on HOMA-IR.

Thirty articles that reported 32 effect sizes were included for the meta-analysis that evaluated the effect of RV on glucose levels, 24 publications with 26 effect sizes for insulin, 16 articles with 17 effect sizes of RV on HbA1c, and 26 studies with 28 effect sizes on HOMA-IR. There was a statistically significant decrease of glucose (−5.24 mg/dL, *p* = 0.002; Figure 3) and insulin levels (−1.23 mIU/L, *p* = 0.0003; Figure 4). HbA1c (Figure 5) and HOMA-IR (Figure 6) did not show significant changes. Due to the considerable heterogeneity of the included studies, sub-analyzes were performed (Table 2). Sub-analysis by dose revealed that glucose levels improve significantly after the administration of 500–1000 mg/day of RV (−7.54 mg/dL, *p* = 0.002), while insulin improves with doses lower than 500 mg/day (−1.43 mIU/L, *p* = 0.01) and with doses greater than 1000 mg/day (−2.12 mIU/L, *p* = 0.03). HbA1c and HOMA-IR remained unchanged after sub-analysis by dose.

In the sub-analysis by health status, we found that the four parameters evaluated improve significantly after the administration of RV in subjects with T2DM (effect size on glucose = −13.36 mg/dL, *p* = 0.0007; effect size on insulin = −0.94 mIU/L, *p* = 0.007; effect size on HbA1c = −0.22%, *p* = 0.02; effect size of HOMA-IR = −0.83, *p* = 0.04). The sub-analysis by duration (<3 months or ≥3 months) revealed that glucose levels decrease significantly after RV administration for <3 months (−5.29 mg/dL, *p* = 0.008). Insulin levels improve regardless of the duration of the intervention (effect size at <3 months = −0.93 mIU/L, *p* = 0.002; effect size at ≥3 months = −1.65 mIU/L, *p* = 0.03). HbA1c improves significantly (effect size = −0.29%, *p* = 0.006) in interventions lasting ≥3 months. HOMA-IR is not significantly modified, regardless of the duration of the intervention.

Finally, the sub-analysis by age revealed that glucose, insulin, and HbA1c levels significantly improve in subjects aged 45 to 59 years who use RV (effect size on glucose = −11.04 mg/dL, *p* = 0.007; effect size on insulin = −0.97, *p* = 0.02; effect size on HbA1c = −0.34%, *p* = 0.002). However, HOMA-IR exhibited no significant changes.

## 4. Discussion

Currently, the incidence of NCDs, such as obesity, diabetes, cardiovascular diseases (CVD), and metabolic syndrome (MS), is increasing and according to the World Health Organization (WHO), is the main cause of death worldwide. The uncontrolled increase in NCDs is related to unhealthy lifestyles, such as diets rich in carbohydrates and fat, sedentary lifestyles, and tobacco and alcohol consumption [50,51,52,53,54]. For this reason, the main strategies applied for the prevention and control of these pathologies focus on achieving a change in lifestyles and improving therapeutic adherence in the population at risk [55,56,57]. However, it is well-known that the proposed strategies have not been entirely successful and the search for new therapeutic agents has been necessary, among which nutraceuticals stand out. These compounds have aroused great interest among the scientific community, including phenolic acids, stilbenes, flavonoids, lignans, and curcuminoids, which have been the object of multiple investigations aimed at understanding their role in preventing diseases and increasing longevity [58,59,60]. In this sense, RV has been widely studied. Some research suggests that its use is associated with a lower incidence and better control of a wide variety of NCDs. This occurs due to the antioxidant capacity of RV and its interaction with cell signaling pathways for the modulation of gene expression. However, other investigations show the lack of a therapeutic effect of this nutraceutical [19,22,23,24,25,27,29,33,34,35,61,62,63]. This means that researchers need to continue conducting clinical trials and analyzing existing ones to identify the efficacy and safety of RV as a complementary treatment for NCDs.

This meta-analysis contains 30 articles that study the effects of RV supplementation vs. a placebo on glucose, insulin, HbA1c, and insulin resistance (measured by the HOMA-IR index). These biochemical parameters are important for evaluating the prevention and control of metabolic diseases such as T2DM, obesity, nonalcoholic fatty liver, and MS. For this reason, they are the main biomarkers of outcome in most clinical trials evaluating the effectiveness of RV.

Our global results show that RV supplementation vs. a placebo decreases glucose and insulin levels, but has no therapeutic effect on HbA1c and HOMA-IR, which is contrary to what was found in the meta-analysis by Hausenblas et al. [64], who observed a significant decrease in HbA1c, without a considerable effect on glucose levels. In addition to this, in the study carried out by Jeyaraman et al. [65], they found that RV did not significantly improve HbA1c, glucose, and insulin levels.

Among the biochemical parameters most used in research, due to their reliability in evaluating the therapeutic efficacy of different nutraceuticals in the control of metabolic diseases, are HbA1c, insulin resistance (calculated by the HOMA-IR index), fasting glucose, and insulin. On the one hand, HbA1c is formed when glucose binds to an amino group of the β chain of hemoglobin through a non-enzymatic reaction that is influenced by the concentration of glucose in the blood, so that a state of hyperglycemia is manifested as a high percentage of HbA1c [66]. On the other hand, it is known that insulin is the most important regulator in glucose and lipid metabolism, so insulin resistance is a distinctive feature of obesity, T2DM, and cardiovascular diseases [67].

The evidence from our meta-analysis shows that RV consumption does not improve HbA1c and insulin resistance, since, in most of the included studies, there were no significant changes in these parameters. Given the above, our results suggest that RV administration is not effective for prolonged glycemic control (around 90–120 days). However, there is considerable heterogeneity between the studies, which is attributed to the wide variation of RV dosage, duration of administration, and number of participants. Furthermore, some studies were at risk of bias in selection and blinding, due to the open and single-blind design [23,26,29,30,39,43,46,48].

Considering the general results and the influence of heterogeneity, a subgroup analysis was performed, stratifying the publications included by dose, health status, duration of intervention, and age of the participants.

### 4.1. Sub-Analysis by RV Dosage

After performing the stratified analysis by dose, a positive and statistically significant effect of RV on glucose levels was found at doses of 500–1000 mg/day, while the effect of RV on insulin was significant after consuming doses of less than 500 mg/day and greater than 1000 mg/day. In the systematic review and meta-analysis carried out by Zhu et al. [68], they found that, at doses of less than 100 mg/day, there are no changes in glucose levels, but higher doses (even 1 g) are capable of decreasing glucose levels, which partially coincides with our results. This is due to the fact that Zhu et al. only included subjects with T2DM, while in our study, subjects with and without T2DM were included. It has been shown that the efficacy of RV may differ according to the administered dose, because the molecular target changes. In addition, it has been proposed that RV could have a dose–response effect (hormesis), so, at low doses, it triggers a stimulating response of some metabolic pathways, and at high doses, it causes the inhibition of the same pathways [69].

SIRT1 is known to play an important role in AMPK activation to improve mitochondrial function and stimulate glucose utilization, as well as protect cells against metabolic decline. In this regard, both in vitro and in vivo studies have shown that moderate doses of RV activate SIRT1 and this, in turn, activates AMPK. In contrast, high doses activate AMPK independently of SIRT1, but do not improve mitochondrial function or protect against metabolic deterioration [70]. It has also been observed that in murine models, low doses of RV improve the insulin sensitivity and decrease its secretion by parts of the pancreatic β cells in the long term, while high doses have the same effect in the short term; however, high doses of RV cause nephrotoxicity [71].

In our meta-analysis, we found that high and low doses of RV exert similar effects on insulin levels. However, the variability in the duration of the interventions and in the health conditions of the participants does not allow us to establish if this result is due to the biological effects of RV occurring in a dose-dependent manner or a consequence of the metabolic conditions of cells, since, depending on the cellular needs, RV activates different molecules and signaling pathways, which translates into different biological effects [7,8]. In addition, it should be emphasized that changes in insulin levels after RV administration, although statistically significant, do not necessarily represent a clinically important change. Due to this, it is necessary to carry out more research on the biological effects of RV to determine if these are presented in a dose-dependent manner in humans, since, so far, many of the results in animal models have not been reproduced in humans. For this reason, it is very difficult to propose a therapeutic dose of RV.

Regarding the insulin resistance markers (HOMA-IR) and HbA1c, in this review, no significant changes were observed in these parameters, which, in addition to being consistent among most of the publications included, coincides with that reported by Zhu et al. [68].

### 4.2. Sub-Analysis by Health Condition

According to the analysis by the presence or absence of T2DM, we observed that RV consumption had a positive effect on the four measured parameters (glucose, insulin, HOMA-IR, and HbA1c), in favor of the subjects with T2DM, which was consistent with the majority of the results from clinical trials conducted in diabetic subjects that were included in the meta-analysis (Abdollahi et al.; Bhatt et al.; Hoseini et al.; Javid et al.; Khodabandenlhoo et al.; Movahed et al.; and Sattarinezhad et al.) [18,22,35,36,38,41,44]. They observed a significant decrease in glycemic control markers after RV consumption in diabetic subjects. These results are consistent with the meta-analysis by Liu et al. [72], where they found that RV consumption significantly reduced glucose, insulin, insulin resistance, and HbA1c levels in participants with T2DM.

The hypoglycemic effect of RV has been attributed to its antioxidant and anti-inflammatory properties. It is known that molecular targets include SIRT1, AMPK, nuclear factor kappa β, and transcription factor Nrf2, among others [73]. It has been demonstrated in several in vitro experiments and in vivo in diabetic animal models that RV increases glucose uptake, utilization, and storage, at the same time that it restores insulin signaling pathways and increases its sensitivity [74,75,76]. The proposed mechanisms are the following:

- Increases the expression of GLUT4 (an insulin-dependent glucose transporter) and improves glucose uptake;
- Activation of SIRT1, which modulates different metabolic pathways, as follows: (i) It deacetylates the FOXO 1 protein, inhibiting its activity and suppressing the apoptosis of pancreatic β cells; (ii) it reduces the expression of the nuclear factor kappa β, which translates into a decrease in the activity of inflammation markers and oxidative stress, responsible for the production of advanced glycation end products (AGE); (iii) it activates AMPK, which regulates various intracellular processes, such as energy metabolism, mitochondrial functions, and cellular homeostasis. AMPK inactivity is correlated with insulin resistance and tissue damage caused by hyperglycemia; and (iv) it activates FOXO 3 expression, thereby suppressing the production of reactive oxygen species and improving regulation in manganese superoxide dismutase (MnSOD) expression;
- Decreases the expression of the AGE receptor (RAGE) that contributes to insulin resistance by modifying its receptor proteins, by phosphorylating the serine/threonine segment, causing insulin resistance. Therefore, the decrease in the production and activity of AGE improves insulin signaling;
- Activation of factor Nrf2, which is a transcription factor that coordinates the activation of a wide range of genes of antioxidant systems, thereby increasing the activity of the antioxidant enzymes glutathione peroxidase (GPx), glutathione reductase (GR), superoxide dismutase (SOD), and catalase.

In the meta-analysis carried out by Liu et al. [72], non-diabetic subjects who consumed RV did not show a significant decrease in the glycemic control parameters. In this meta-analysis, we found similar results. The same has been reported in other investigations for healthy animal models [76]. In these studies, it was has been observed that RV administration does not have a significant effect on glucose, the lipid profile, and the insulin sensitivity, although the cellular mechanisms are not entirely clear [9,11]. These results can be explained considering that, in normal physiological conditions, glucose and insulin concentrations are in an acceptable range. Therefore, there are no metabolic alterations and RV consumption does not activate the molecular targets or metabolic pathways that are affected due to the presence of T2DM. In this sense, the results suggest that RV does not cause hypoglycemia in healthy people, although more quality clinical trials are required to evaluate the effects of RV consumption in healthy people.

### 4.3. Sub-Analysis by Duration of Intervention

Analysis by duration of the intervention (studies with an intervention <3 months and studies with an intervention ≥3 months) revealed a positive effect on glucose when the intervention was less than three months. HbA1c showed a significant decrease when the intervention had a duration of more than three months, while the effect on insulin was positive in both interventions (<3 months and ≥3 months). However, the HOMA-IR index had no significant effect regarding the duration of the intervention.

The discrepancy in glucose and HbA1c results is due to the serum glucose levels reflecting a very short period of glucose metabolism and being influenced by diet in the short term. In contrast, HbA1c reflects glucose metabolism for a period ranging from 90 to 120 days, which is why it is considered a highly reliable marker of long-term glycemic control. In this regard, the results of different clinical trials included in this meta-analysis show that the intervention time plays an important role in glycemic control. Abdollahi et al. [18] observed that the administration of 1 g/day of RV for 8 weeks is not enough to have a positive effect on HbA1C, despite lowering glucose levels, as did Thazhath et al. [47], who reported that 5 weeks of treatment with 1 g/day of RV has no effect on HbA1c levels in diabetic patients. On the other hand, Bhatt et al. [22] reported that 3 months of supplementation with 250 mg/day of RV significantly reduces HbA1c, while Sattarinezhad et al. [44] found that 500 mg/day of RV for 3 months triggers a significant decrease in HbA1C, insulin, and the HOMA-IR index.

Our results are consistent with the study by Timmers et al. [77] carried out in obese subjects. This study reported that RV consumption for a period of 30 days improves glucose homeostasis and insulin resistance because it mimics the effects of caloric restriction. Meanwhile, the meta-analysis carried out by Guo et al. [78], who evaluated the effects of VR intervention on risk factors for NCDs, showed that a 3-month intervention significantly reduces low-density lipoproteins (LDL-cholesterol) and HbA1c levels.

### 4.4. Sub-Analysis by Age

Three groups were formed according to the age of participants: Those (i) under 45 years old; (ii) from 45 to 59 years old; and (iii) over 60 years old. Significant changes in favor of RV were only presented for glucose, insulin, and HbA1c levels in the studies that included subjects aged 45 to 59 years, while the HOMA-IR index did not have significant changes in any group.

These results are in contrast to the findings of Crandall et al. [79] and Witte et al. [80], who found that RV administration in older adults improves the insulin sensitivity, plasma glucose, and glucose metabolism. However, in the clinical trials included in our meta-analysis, which were conducted in subjects under 45 years of age (Asghari et al.; Bo et al.; Godínez-Salas et al.; Poulsen et al. [19,25,33,43]), it was observed that glycemic control markers did not change. Moreover, among clinical trials with people older than 60 years, only Hoseini et al. [35] reported a significant change in glucose levels after an intervention with 500 mg/day of RV for 4 weeks. Most of the studies where the age of the participants ranged between 45 and 59 years found significant changes in the biomarkers of glycemic control, except those with low doses of RV (Kantartzis et al. [37]) or short intervention periods (Dash et al. [29]).

RV is a nutraceutical widely studied for the control of metabolic diseases due to its antioxidant and anti-inflammatory properties. Its role has been demonstrated in preclinical studies, but its effects in humans are controversial. This is probably due to its unfavorable pharmacokinetics and its low bioavailability, which could be influenced by the intestinal microbiota [81,82]. In addition, the genetic influence is an important factor for the individual response to RV [83]. RV has been shown to activate the expression of SIRT1, which is a histone deacetylase that plays a crucial role in glucose metabolism, lipids, the inflammatory process, and antioxidant defenses [74,75,84]. In vivo investigations have indicated that, in aging, the activity of SIRT1 is decreased [85], which could cause a poor response of the body to the administration of RV in older adults.

The controversy about the biological effects of RV in humans justifies the continuity of research, and it is necessary to know the efficacy and safety of RV in the prevention and treatment of high prevalence metabolic diseases, most of which are related to oxidative stress and inflammatory process. Another important factor to elucidate is the metabolic pathways that it activates and how age, health status, dose, and time of treatment influence these.

### 4.5. Limitations

This meta-analysis was not registered in PROSPERO; however, the guidelines established in PRISMA were followed.

## 5. Conclusions

The findings of this meta-analysis suggest that RV significantly improves glucose and insulin levels in subjects with T2DM and aged 45–59 years, regardless of the duration of the intervention. Meanwhile, HbA1c improves significantly with interventions whose duration is greater than 3 months. Nevertheless, the insulin resistance measured by HOMA-IR does not display significant changes after RV administration. Regarding the dose used, the results do not allow a therapeutic dose to be suggested. Therefore, more clinical trials are required to identify how the RV dosage, duration of the interventions, health status, and age of the subjects influence the biological effects of RV, since, due to the heterogeneity presented by the available publications, the results are not conclusive. In this sense, it would be convenient to carry out studies that compare the effects of RV in young vs. old adults, in addition to studies comparing small vs. large doses. Long-term follow-up studies (>12 months) with intermediate measurements in the short and medium term (1, 3, 6, and 12 months, for example) could also be carried out, in order to observe and compare the effects of RV at different doses and durations. Finally, it is recommended that future clinical trials analyze and compare the results of subjects with different health conditions, such as diabetes, hypertension, dyslipidemias, and metabolic syndrome, since this will allow more clarity about the hypoglycemic efficacy of RV, as well as the proposal of a therapeutic dose, depending on the patient’s conditions (age and health status). In addition to this, long-term research will be useful for obtaining data about the safety of this compound over long periods of time.

## Figures and Tables

**Table 1:** Characteristics of clinical trials included in the review.

First Author (Year)	Study Design	RV Dosage	Duration	Subjects	Age	Evaluated Parameters	Findings
**Abdollahi et al. (2019)** [18]	RCT double-blind	1 g/day	8 weeks	71 subjects with T2DM and overweight	50 ± 7	Glucose and insulin levels, HbA1c, HOMA-IR, HOMA-β, QUICKI	Significant decrease in glucose (*p* = 0.03) and insulin (*p* = 0.02), improvement in HOMA-IR (*p* = 0.01) and QUICKI (*p* = 0.008). No significant changes in HOMA-β and HbA1c after resveratrol administration
**Asghari et al. (2018)** [19]	RCT double-blind	600 mg/day	12 weeks	75 subjects with fatty liver	40 ± 7	Glucose and insulin levels HOMA-IR	Non-significant changes in the glycemic parameters evaluated
**Banaszewska et al. (2016)** [20]	RCT double-blind	1.5 g/day	3 months	30 women with POS	27 ± 1	Fasting blood glucose, insulin sensitivity index	Significant decrease in insulin levels (38%, *p* = 0.007) and increase in the insulin sensitivity index (66%, *p* = 0.04)
**Bashmakov et al. (2014)** [21]	RCT parallel-blind	100 mg/day	2 months	24 patients with diabetic food	56 ± 9	Glucose and insulin levels, HOMA-IR	Tendency of a decrease of glucose levels in both study groups. No changes in insulin levels and HOMA-IR
**Bhatt et al. (2012)** [22]	RCT open-label	250 mg/day	3 months	57 subjects with T2DM	57 ± 9	Fasting blood glucose and HbA1c	Significant decrease in HbA1c levels (*p* < 0.05) after resveratrol administration
**Bhatt et al. (2013)** [23]	RCT open-label	250 mg/day	6 months	57 subjects with T2DM	57 ± 9	HbA1c and glucose levels	Non-significant decrease in HbA1c and glucose levels after intervention
**Bo et al. (2016)** [24]	RCT double-blind	40, 500 mg/day	6 months	179 subjects with T2DM	65 ± 8	Glucose and insulin levels, HOMA-IR, HbA1c, C-peptide	Non-significant differences between the study groups after intervention
**Bo et al. (2013)** [25]	RCT double-blind crossover	500 mg/day	4 weeks	49 healthy smokers	35 ± 9	Glucose and insulin levels HOMA-IR	Non-significant changes after resveratrol intervention
**Brasnyó et al. (2011)** [26]	RCT double-blind	10 mg/day	4 weeks	19 men with T2DM	55 ± 9	Insulin levels, HOMA-IR, HOMA-β	No changes in insulin and HOMA-β levels, tendency of a decrease of HOMA-IR in the experimental group
**Chachay et al. (2014)** [27]	RCT double-blind	3 g/day	8 weeks	20 men with NAFLD	49 ± 12	HOMA-IR, glucose and insulin levels	Resveratrol did not improve glucose, insulin, and HOMA-IR levels
**Chen (2015)** [28]	RCT double-blind	600 mg/day	3 months	57 subjects with NAFLD	44 ± 10	Glucose, insulin, C-peptide and HOMA-IR	Significant decrease in glucose (*p* = 0.001) and HOMA-IR (*p* = 0.016). No significant changes in insulin and C-peptide levels
**Dash et al. (2013)** [29]	RCT double-blind crossover	1–2 g/day	2 weeks	8 overweight and obese subjects	46 ± 3	Glucose and insulin levels, HOMA-IR	Non-significant changes in evaluated parameters after resveratrol administration
**De Ligt et al. (2020)** [30]	RCT double-blind	150 mg/day	6 months	41 overweight individuals	62 ± 1	Glucose and insulin levels, HbA1c	Non-significant differences in glucose and insulin levels. Significant decrease in HbA1c (*p* = 0.007) after resveratrol administration
**De Ligt et al. (2018)** [31]	RCT double-blind crossover	150 mg/day	4 weeks	13 men at high risk of T2DM	66 ± 4	Glucose and insulin levels, HbA1c	Non-significant differences between the study groups after resveratrol administration
**Faghihzadeh et al. (2015)** [32]	RCT double-blind	500 mg/day	12 weeks	50 subjects with NAFLD	45 ± 10	Glucose and insulin levels, HOMA-IR, HOMA-β, QUICKI	Non-significant changes after the resveratrol intervention
**Godínez-Salas et al. (2018)** [33]	RCT double-blind	150 mg/day	3 months	42 subjects with MS	43 ± 1	Glucose and insulin levels	Non-significant changes in glucose and insulin levels after resveratrol intervention
**Goh et al. (2014)** [34]	RCT double-blind	3 g/day	12 weeks	10 subjects with T2DM	56 ± 6	Glucose and insulin levels, HbA1c, HOMA-IR	Tendency of a decrease in HbA1c, no significant changes in insulin sensitivity
**Hoseini et al. (2019)** [35]	RCT double-blind	500 mg/day	4 weeks	56 subjects with T2DM and CD	62 ± 9	Glucose and insulin levels, HOMA-IR, QUICKI	Significant decrease in glucose, insulin (*p* = 0.01), and HOMA-IR (*p* = 0.001); QUICKI increase (*p* = 0.02)
**Javid et al. (2016)** [36]	RCT double-blind	480 mg/day	4 weeks	43 subjects with T2DM and CP	50 ± 8	Glucose and insulin levels, HOMA-IR	Significant decrease in insulin and HOMA-IR levels (*p* < 0.05), non-significant decrease in glucose levels after resveratrol intervention
**Kantartzis et al. (2018)** [37]	RCT double-blind	150 mg/day	12 weeks	105 overweight and obese subjects	48 ± 13	Fasting blood glucose, HbA1c, HOMA-IR	No significant changes in evaluated parameters
**Khodabandenlhoo et al. (2018)** [38]	RCT double-blind	800 mg/day	2 months	45 subjects with T2DM	57 ± 9	Glucose and insulin levels, HbA1c, HOMA-IR, HOMA-β	Significant decrease in glucose levels (*p* < 0.05) in experimental group. No changes in HbA1c. No significant changes in insulin, HOMA-IR, and HOMA β levels
**Kjaer et al. (2017)** [39]	RCT double-blind	1,501,000 mg/day	16 weeks	66 subjects with MS	50 ± 1	Glucose and insulin levels, HOMA-IR	No change in glucose and insulin concentrations, no change in HOMA-IR
**Méndez-del Villar et al.(2014)** [40]	RCT double-blind	1.5 g/day	3 months	21 subjects with MS	40 ± 5	AUC of glucose and insulin, insulin index	Significant decrease in insulin AUC and insulin index (*p* < 0.05)
**Movahed et al. (2013)** [41]	RCT double-blind	1 g/day	45 days	64 subjects with T2DM	52 ± 7	Glucose and insulin levels, HOMA-IR, HOMA-β	Significant decrease (*p* < 0.05) in glucose, insulin, and HbA1c levels. Improvement of HOMA-IR and HOMA β in experimental group
**Pollack et al. (2017)** [42]	RCT double-blindcrossover	2 g/day	6 weeks	30 overweight older adults with impaired glucose tolerance	67 ± 7	Glucose and insulin levels, AUC of glucose and insulin, HbA1c, HOMA-IR	No effect of resveratrol on glycemic parameters
**Poulsen et al. (2013)** [43]	RCT double-blind	500 mg/day	4 weeks	24 obese subjects	39 ± 3	Glucose and insulin levels, HbA1c, HOMA-IR	No changes in evaluated parameters
**Sattarinezhad et al. (2019)** [44]	RCT double-blind	500 mg/day	3 months	60 subjects with T2DM and albuminuria	57 ± 9	Glucose and insulin levels, HbA1c, HOMA-IR	Significant decrease in evaluated parameters in experimental group (*p* < 0.05)
**Seyyedebrahimi et al. (2018)** [45]	RCT double-blind	800 mg/day	8 weeks	46 subjects with T2DM	58 ± 6	Glucose and insulin levels, HbA1c, HOMA-IR	No significant changes after resveratrol intervention
**Thaung et al. (2020)** [46]	RCT double-blind	150 mg/day	12 months	129 postmenopausal women	64 ± 1	Glucose and insulin levels, HOMA-IR	No significant changes in evaluated parameters
**Thazhath et al. (2016)** [47]	RCT double-blindcrossover	1 g/day	5 weeks	14 subjects with T2DM	68 ± 2	Glucose and HbA1c	No significant changes in glucose and HbA1c levels
**Timmers et al. (2016)** [48]	RCT double-blindcrossover	150 mg/day	4 weeks	16 subjects with T2DM	64 ± 4	Glucose and insulin levels, insulin sensitivity, HbA1c	No effect of resveratrol on glycemic parameters
**Van der Made et al.(2015)** [49]	RCT double-blindcrossover	150 mg/day	4 weeks	45 overweight and obese subjects	61 ± 7	Glucose and insulin levels, HOMA-IR	No changes in HOMA-IR and insulin levels. Glucose levels showed a tendency to decrease

Abbreviations: AUC, area under curve; CD, coronary disease; CP, chronic periodontitis; HbA1c, glycated hemoglobin; HOMA-β, insulin resistance of pancreatic cell β; HOMA-IR, insulin resistance; MS, metabolic syndrome; NAFLD, non-alcoholic fatty liver disease; POS, polycystic ovary syndrome; RCT, randomized clinical trials; T2DM, type 2 diabetes mellitus; and QUICKI, Quantitative Insulin Sensitivity Check Index.

**Table 2:** Subgroup analysis conducted to evaluate the hypoglycemic effect of resveratrol.

Subgroup	No. of Trials	Effect Size	95% CI	*p* Value	Heterogeneity (I^2^)	*p* Value for I^2^
**Glucose**						
**Resveratrol dosage (I^2^ = 76%; *p* = 0.01)**						
**<500 mg/day**	13	−5.40	−11.29, 0.49	0.07	97%	0.00001
**500–1000 mg/day**	13	−7.54	−12.29, −2.79	0.002	89%	0.00001
**>1000 mg/day**	6	0.82	−2.78, 4.42	0.66	51%	0.00001
**Health status (I^2^ = 91%; *p* = 0.0008)**						
**With T2DM**	15	−13.36	−21.09, −5.63	0.0007	96%	0.00001
**Without T2DM**	17	0.18	−1.52, 1.89	0.83	67%	0.0001
**Duration (I^2^ = 0%; *p* = 0.89)**						
**<3 months**	15	−5.29	−9.20, −1.39	0.008	81%	0.00001
**≥3 months**	17	−4.83	−9.75, 0.09	0.05	96%	0.00001
**Age (I^2^ = 66%; *p* = 0.05)**						
**<45 years**	6	0.08	−4.08, −4.24	0.97	79%	0.0002
**45–59 years**	16	−11.04	−19.0, −3.07	0.007	96%	0.00001
**≥60 years**	10	−2.06	−4.39, 0.27	0.08	73%	0.0001
**Insulin**						
**Resveratrol dosage (I^2^ = 0%; *p* = 0.44)**						
**<500 mg/day**	9	−1.43	−2.53, −0.32	0.01	90%	0.00001
**500–1000 mg/day**	12	−0.78	−1.85, 0.30	0.16	83%	0.00001
**>1000 mg/day**	5	−1.23	−1.90, −0.57	0.03	73%	0.006
**Health status (I^2^ = 0%; *p* = 0.57)**						
**With T2DM**	12	−0.94	−1.62, −0.25	0.007	77%	0.00001
**Without T2DM**	14	−1.39	−2.80, 0.01	0.05	91%	0.00001
**Duration (I^2^ = 0%; *p* = 0.38)**						
**<3 months**	13	−0.93	−1.53, −0.33	0.002	69%	0.0001
**≥3 months**	13	−1.65	−3.15, −0.16	0.03	93%	0.00001
**Age (I^2^ = 42%; *p* = 0.18)**						
**<45 years**	5	−3.60	−7.65, 0.46	0.08	96%	0.00001
**45–59 years**	13	−0.97	−1.82, −0.12	0.02	82%	0.00001
**≥60 years**	8	−0.32	−1.01, 0.36	0.35	57%	0.02
**HbA1c**						
**Resveratrol dosage (I^2^ = 0%; *p* = 0.56)**						
**<500 mg/day**	7	−0.20	−0.42, 0.02	0.08	99%	0.00001
**500–1000 mg/day**	8	−0.06	−0.21, 0.10	0.48	69%	0.002
**>1000 mg/day**	2	−0.25	−1.18, 0.69	0.61	68%	0.08
Health status (I^2^ = 87%; *p* = 0.005)						
**With T2DM**	12	−0.22	−0.40, −0.04	0.02	97%	0.00001
**Without T2DM**	5	0.05	−0.01, 0.10	0.11	0%	0.78
Duration (I^2^ = 0%; *p* = 0.38)						
**<3 months**	9	0.02	−0.08, 0.13	0.66	64%	0.004
**≥3 months**	8	−0.29	−0.50, −0.08	0.006	98%	0.00001
Age (I^2^ = 85%; *p* = 0.001)						
**<45 years**	1	0.05	−0.04, 0.14	0.29	---	---
**45–59 years**	9	−0.34	−0.54, −0.13	0.002	98%	0.00001
**≥60 years**	6	0.07	0, 0.15	0.05	0	0.48
**HOMA-IR**						
**Resveratrol dosage (I^2^ = 0%; *p* = 0.62)**						
**<500 mg/day**	11	−0.22	−0.55, 0.11	0.19	57%	0.01
**500–1000 mg/day**	12	−0.60	−1.44, 0.24	0.16	97%	0.00001
**>1000 mg/day**	5	−0.22	−0.74, 0.30	0.42	46%	0.11
**Health status (I^2^ = 64%; *p* = 0.10)**						
**With T2DM**	12	−0.83	−1.68, −0.02	0.04	96%	0.00001
**Without T2DM**	16	−0.08	−0.33, 0.17	0.54	55%	0.004
**Duration (I^2^ = 0%; *p* = 0.83)**						
**<3 months**	14	−0.36	−1.11, 0.39	0.35	96%	0.00001
**≥3 months**	14	−0.45	−0.91, 0.00	0.05	83%	0.00001
**Age (I^2^ = 0%; *p* = 0.70)**						
**<45 years**	5	−0.57	−1.19, 0.04	0.07	68%	0.01
**45–59 years**	14	−0.39	−1.25, 0.47	0.38	96%	0.00001
**≥60 years**	9	−0.28	−0.60, 0.03	0.08	55%	0.02

Abbreviations: CI, confidence interval; HbA1c, glycated hemoglobin; HOMA-IR, insulin resistance (homeostatic model); and T2DM, type 2 diabetes mellitus.

## Appendix A. Studies Excluded from the Systematic Review and Meta-Analysis

**Study**	**Reason for Exclusion**
Abdollahi, et al. BMJ. 2019;9:e026337, doi:10.1136/ bmjopen-2018-026337	It is a protocol
Asghari, et al. Adv Phar Bull. 2018, 8(2), 307–317, doi:10.15171/apb.2018.036	They do not evaluate glycemic parameters
Bo, et al. Acta Diabetol. 2018;55:331–3402018, doi:10.1007/s00592-017-1097-4	They do not report pre- and post-treatment means of glycemic parameters
Brenjian, et al. Am J Reprod Immunol. 2020;83:e13186, doi:10.1111/aji.13186	They do not evaluate glycemic parameters
Cao, et al. Exp Ther Med. 2018; 15: 576–584, doi:10.3892/etm.2017.5400	They do not evaluate glycemic parameters
Crandall, et al. J Gerontol A Biol Sci Med Sci. 2012;67:1307–1312, doi:10.1093/gerona/glr235	It is a pilot study
Foroghi, et al. IJEM. 2018;20:169-176.	Language other than English
Gospin, et al. J Investig Med 2016;64:800–825, doi:1 0.1136/jim-2016-000080.35	Only abstract available
Huhn, et al. 2018. NeuroImage Doi:10.1016/j.neuroimage.2018.03.023	They use a combination of resveratrol with quercetin
Kjaer, et al. The Prostate. 2015;75:1255–1263, doi:10.1002/pros.23006	They do not evaluate glycemic parameters
Knop, et al. Diabet Med. 2013;61:1886 Doi:10.1111/dme.12231	They do not evaluate glycemic parameters
Köbe, et al. Front. Neurosci. 2017. 11:105. Doi:10.3389/fnins.2017.00105	They use a combination of resveratrol with quercetin
Konings, et al. Int J Obes. 2014;38:470–473. Doi:10.1038/ijo.2013.155	They do not evaluate glycemic parameters
Korsholm, et al. Int. J. Mol. Sci. 2017, 18, 554; doi:10.3390/ijms18030554	They do not evaluate glycemic parameters
Maginley, et al. J Investig Med. 2019;67:793, doi:10.1136/jim-2019-001036.20	Only abstract available
Mahmood, et al. J. Pharm Sci Res. 2018;10(5):999–1005.	They do not evaluate glycemic parameters
Milton-Laskibar, et al. IUBMB. 2016, doi:10.1002/biof.1347	Study carried out on animals
Most et al. Am J Clin Nutr 2016;104:215–27, doi:10.3945/ajcn.115.122937	They use a combination of resveratrol with epigallocatechin
Ornstrup, et al. J Clin Endocrinol Metab. 2014;99:4720–4729, doi:10.1210/jc.2014-2799	They do not evaluate glycemic parameters
Pankaj, et al. Biochem Bioph Res Co. 2015. 10.1016/j.bbrc.2015.10.126	Study carried out on animals
Poulsen, et al. Diabetes Obes Metab. 2018;20:2504–2509, doi:10.1111/dom.13409	They do not report pre- and post-treatment means of glycemic parameters
Theodotou, et al. Exp Ther Med. 2018; 18: 559–565, doi:10.3892/etm.2019.7607	The comparison group is not a placebo
Van der Made, et al. Nutrients. 2017;9,596, doi:10.3390/nu9060596	They do not evaluate glycemic parameters
Vatavuk-Serrati, et al. Rev Soc Cardiol Estado de São Paulo-Supl-2019;29(1):88–93	Language other than English
Voduc, et al. Appl. Physiol. Nutr. Metab. 2014. 39:1183–1188. Doi.org/10.1139/apnm-2013-0547	It is a pilot study
Walker, et al. J Clin Transl Res. 2019;4:122–135. Doi:10.18053/jctres.04.201802.004	It is a pilot study
Wicklow, et al. Biochem Cell Biol. 2015; 93: 1–9, doi:/10.1139/bcb-2014-01362015	It is a protocol
Witte, et al. J Neurosci. 2014. 4(23):7862–7870, doi:10.1523/JNEURISCI.0385-14.2014	They use a combination of resveratrol with quercetin
Wong, et al. Nutr Metabol Cardiovasc Dis. 2016, doi:10.1016/j.numecd.2016.03.003	They use a single dose of RV
Wong, et al. Nutrients. 2016, 8, 425, doi:10.3390/nu8070425	They use a single dose of RV
